# *In vivo* molecular neuroimaging of glucose utilization and its association with fibrillar amyloid-β load in aged APPPS1-21 mice

**DOI:** 10.1186/s13195-015-0158-6

**Published:** 2015-12-15

**Authors:** Ann-Marie Waldron, Cindy Wintmolders, Astrid Bottelbergs, Jonathan B. Kelley, Mark E. Schmidt, Sigrid Stroobants, Xavier Langlois, Steven Staelens

**Affiliations:** Molecular Imaging Center Antwerp, University of Antwerp, Campus Drie Eiken – UC, Universiteitsplein 1, 2610, Wilrijk, Antwerp Belgium; Neuroscience Research & Development, Janssen Pharmaceutica NV, Beerse, Belgium; Nuclear Medicine Department, University Hospital Antwerp, Antwerp, Belgium

## Abstract

**Introduction:**

Radioligand imaging is a powerful *in vivo* method to assess the molecular basis of Alzheimer’s Disease. We therefore aimed to visualize the pathological deposition of fibrillar amyloid-β and neuronal dysfunction in aged double transgenic mice.

**Methods:**

Using non-invasive positron emission tomography (PET) we assessed brain glucose utilization with [^18^F]FDG and fibrillar amyloidosis with [^11^C]PiB and [^18^F]AV45 in 12 month old APPPS1-21 (*n* = 10) mice and their age-matched wild-type controls (*n* = 15). PET scans were analyzed with statistical parametric mapping (SPM) to detect significant differences in tracer uptake between genotypes. After imaging, mice were sacrificed and *ex vivo* measures of amyloid-β burden with immunohistochemistry as well as glucose utilization with [^14^C]-2DG autoradiography were obtained as gold standards.

**Results:**

Voxel-wise SPM analysis revealed significantly decreased [^18^F]FDG uptake in aged APPPS1-21 mice in comparison to WT with the thalamus (96.96 %, maxT = 3.35) and striatum (61.21 %, maxT = 3.29) demonstrating the most widespread reductions at the threshold of *p* < 0.01. [^11^C]PiB binding was significantly increased in APPPS1-21 mice, most notably in the hippocampus (87.84 %, maxT = 7.15) and cortex (69.08 %, maxT = 7.95), as detected by SPM voxel-wise analysis at the threshold of *p* < 0.01. Using the same threshold [^18^F]AV45 uptake was comparably lower with less significant differences. Compared to their respective *ex vivo* equivalents [^18^F]FDG demonstrated significant positive correlation to [^14^C]2-DG autoradiography (r = 0.67, *p* <0.0001) while [^11^C]PiB and [^18^F]AV45 binding did not correlate to *ex vivo* immunohistochemistry for amyloid-β (r = 0.25, *p* = 0.07 and r = 0.17, *p* = 0.26 respectively). Lastly no correlation was observed between regions of high amyloid burden and those with decreased glucose utilization (r = 0.001, *p* = 0.99).

**Conclusions:**

Our findings support that fibrillar amyloid-β deposition and reduced glucose utilization can be visualized and quantified with *in vivo* μPET imaging in aged APPPS1-21 mice. Therefore, the combined use of [^18^F]FDG and amyloid μPET imaging can shed light on the underlying relationship between fibrillar amyloid-β pathology and neuronal dysfunction.

## Introduction

Nuclear medicine imaging of the human brain afflicted by Alzheimer’s Disease (AD) has progressed over the last decades from non-specific markers of neurodegeneration, such as altered metabolism [1, 2], blood flow [3], and inflammation [4, 5], to disease-specific markers of amyloid-β [6] and tau tangles [7] believed to instigate the pathologic cascade. For diagnostic purposes, the functional metabolic marker [^18^F]fluorodeoxyglucose ([^18^F]FDG) remains the most routinely used positron emission tomography (PET) radioligand [8] but the more novel amyloid-β tracers are increasingly used since their recent clinical approval (Amyvid, Neuraceq, and Vizamyl) and growing availability. In clinical studies the combined use of these tracers allows the non-invasive tracking of AD from the early pathologic events of amyloid-β deposition to the later neurodegenerative mechanisms related to clinical decline and have proved seminal in understanding the natural history of AD.

Given the clinical utility of [^18^F]FDG and amyloid tracers, there is considerable interest in back-translating their success to preclinical investigations. A number of transgenic mouse models of cerebral amyloidosis are readily available and are generally created by the manipulation of the genes involved in amyloid processing [9]. While investigation of these models has led to a greater understanding of amyloid-related disease mechanisms, they are limited by the primary use of *ex vivo* methods to assess brain pathology. The application of small animal PET imaging (μPET) to preclinical research allows for the simultaneous longitudinal monitoring of a number of physiological processes in a subject over time and can thus enhance the translational value of animal studies. Despite having lower affinity for fibrillar amyloid-β in animal models [10, 11], amyloid tracers have successfully monitored progressive amyloidosis in a number of transgenic cerebral amyloidosis models [11–17] and have been shown sensitive enough to detect treatment-induced reductions in plaque load with an anti-amyloid antibody [11] and a γ-secretase inhibitor [18]. Although [^18^F]FDG is a better-established tracer, its ability to detect cerebral hypometabolism in amyloidosis models with μPET is debated. Early investigations of glucose utilization in transgenic models with [^18^F]FDG focused on the high-resolution technique of *ex vivo* autoradiography. With this method a number of models were shown to have reductions in [^18^F]FDG uptake in brain regions with homology to those affected in clinical AD [19–23]. However when applied *in vivo* with μPET scanners the majority of studies have reported either unchanged [24, 25] or increased [^18^F]FDG [26–28] uptake in transgenic models. Given the lower resolution of μPET in comparison to autoradiography, decreased sensitivity may indeed mask small regional decreases in [^18^F]FDG uptake between genotypes [29]. However these discrepancies may also be accounted for by methodological factors [30] in addition to strain differences. More recently *in vivo* hypometabolism in amyloidosis models has been described [31–33].

The assessment of glucose utilization is a necessary adjunct to amyloid imaging to unravel the relationship between amyloid-β and neuronal dysfunction. While amyloid imaging may provide a read-out of target engagement for amyloid targeted therapies, such therapies should also be tested for their ability to prevent loss of neuronal function. Moreover, monitoring therapeutic efficacy with [^18^F]FDG would provide a valuable *in vivo* biomarker for non-amyloid targeted therapies, such as neuroprotective strategies or symptomatic relief. We have previously investigated the use of [^18^F]FDG and the amyloid tracer [^18^F]florbetapir in the double transgenic TASTPM mouse model and demonstrated significantly increased retention of [^18^F]florbetapir and significantly lower [^18^F]FDG uptake in these aged animals [33]. We aimed here to extend our findings using a different double transgenic model of amyloidosis and confirming our *in vivo* findings with *ex vivo* [^14^C]2-DG autoradiography. We chose to investigate APPPS1-21 mice as they have demonstrated synaptic alterations [34, 35] and cognitive impairment [36, 37] and, thus, represent an interesting model for measuring neuronal dysfunction. We additionally utilized [^11^C]PiB and [^18^F]AV45 binding [38] in these mice as an *in vivo* measure of fibrillar amyloid-β burden.

## Methods

### Animals

We employed APPPS1-21 (*n* = 10) double transgenic mice that co-express the human Swedish double APP mutation KM670/671NL and the human mutated PS1 L166P driven by the neuron-specific Thy-1 promoter element. Due to these mutations APPPS1-21 mice undergo accelerated and severe disease pathogenesis. Amyloid-β deposition begins as early as six weeks of age and at later stages these mice have extensive dense core plaque pathology [36]. Wild-type littermates (WT) were used as controls (C57BL6J, *n* = 15) and all animals were female. The animals were kept under environmentally controlled conditions (12 h light/dark cycle, 20–24 °C and 40–70 % relative humidity) in individually ventilated cages with food and water ad libitum. Animals were group-housed and received environmental enrichment. Mice were 12 months old at the time of imaging. The study protocol was approved by the local Animal Experimental Ethical Committee of the University of Antwerp, Belgium (2012–25) where the *in vivo* experiments were performed. All animal studies were ethically reviewed and carried out in accordance with European Directive 86/609/EEC Welfare and Treatment of Animals.

### Tracer radiosynthesis

[^11^C]PiB was synthesized according to the one step method under neutral conditions as described previously [38]. [^18^F]FDG was prepared using a cassette based GE Fastlab synthesis module (GE Healthcare, Diegem, Belgium). [^18^F]-AV45 was synthesized from the tosyloxy precursor AV-105 by modification of the method as described by Yao and coworkers [39].

### PET image acquisition and analysis

Static μPET scans (Siemens Preclinical Solution, Knoxville, TN, USA) were acquired after intravenous tracer injection and a conscious uptake period. For [^18^F]FDG, mice received 18.65 ± 0.62 MBq (45 min uptake, 20 min scan). Prior to scanning, animals were fasted overnight for 8–12 hours and blood glucose levels were monitored by a diagnostic assay. For [^11^C]PiB, mice received 4.28 ± 1.06 MBq (20 min uptake, 30 min scan) while for [^18^F]AV45 mice received 17.8 ± 0.92 MBq (30 min uptake, 20 min scan). Anesthesia was induced by inhalation of isoflurane (5 % for induction, and 2 % for maintenance during preparation and scanning) supplemented with oxygen. The core body temperature of the animals was maintained via a temperature controlled heating pad.

μPET data was reconstructed with two-dimensional ordered subsets expectation maximization (OSEM2D) [40] algorithm using four subsets and 16 iterations following Fourier rebinning (FORE) [41]. The energy and timing window was set to 350–650 keV and 3.432 nsec, respectively. The μPET images were reconstructed on a 128 x 128 x 159 grid with a pixel size of 0.776 mm and a slice thickness of 0.796 mm. Normalization, dead time, randoms, CT-based attenuation and single scatter simulation (SSS) [42] corrections were applied. A five min CT was acquired subsequent to all PET scans using a 220 degree rotation with 12- rotation steps; voltage and amperage were set to 80 keV and 500 uA, respectively.

Volume-of-interest (VOI) and voxel-wise analysis (statistical parametric mapping) methods were performed on reconstructed images using PMOD v3.3 (PMOD technologies, Zurich, Switzerland) and SPM8 (Wellcome Trust Centre, London, UK). Individual PET images were spatially normalized into the space of a predefined mouse brain template [43] by matching the individual animal specific CT to the template CT and applying the same transform to the PET which is by default matched to its CT via the PET/CT scanner hardware. Thereafter, extracerebral activity was removed through the use of a brain mask derived from the Mirrione T_2_ -weighted MR template (*a priori* matched with the same CT template) [43] whereby all voxels outside the brain mask were set to zero intensity and voxels inside the mask were unaltered. For an absolute measure of tracer uptake, normalized images were scaled according to the percent injected dose for [^11^C]PiB and [^18^F]AV45 (tissue uptake[kBq/cc]/injected dose[kBq] * 100) and glucose-corrected percent injected dose for [^18^F]-FDG (tissue uptake[kBq/cc] * blood glucose/injected dose[kBq] *100).

For VOI analysis, processed images were subsequently co-registered with a predefined mouse brain VOI template (encompassing all relevant brain regions) aligned with the aforementioned Mirrione CT/MRI atlas and tracer uptake values were extracted for each delineated VOI. With this approach regional values are quantified as the average uptake over the total number of voxels in a VOI.

For voxel-wise analysis of tracer uptake between genotypes, a two-sample unpaired t-test was applied to normalized and scaled images, with a significance threshold of *p* < 0.01 for all tracers with an extent threshold of 100 voxels (48 mm^3^), uncorrected for multiple comparisons. Quantification of this voxel-wise analysis was investigated by employing two indices: 1) the number of significant voxels in a VOI relative to the total number of voxels (% sign); and 2) the maximal T value (maxT). For qualitative purposes T-maps were smoothed with an isotropic Gaussian kernel with 0.5 mm full width at half maximum (FWHM) subsequent to analysis.

### *Ex vivo* gold standard evaluation

Mice were 13 months old (end of life). Mice were fasted for 12 hr to stabilize plasma glucose levels prior to an intraperitoneal injection of 0.148 MBq (weight adjusted) [^14^C]2-DG. After a conscious uptake period of 45 min, mice were sacrificed by decapitation and the brains rapidly removed. After decapitation plasma glucose was measured by a diagnostic assay.

For autoradiography, the left brain hemisphere was frozen in cooled 2-methylbutane (−30 °C on dry ice) for approximately one min. This hemisphere was then covered in tissue embedding medium which was allowed to cool to −20 °C. Coronal sections (20 μm thick) were sliced in triplicate by a cryostat and thaw mounted on glass microscope slides. The slides were immediately placed on a heating plate (60 °C) for five to ten min to prevent diffusion of [^14^C]2-DG out of the cells. These slices were co-exposed with a commercially available [^14^C] standard to autoradiography film for four days. After the exposure time, the autoradiograms were developed in a dark room. The autoradiograms were quantified with MCID Basic 7.0 (Imaging Research Inc., St.Catherine's, ON, Canada) image analyzer software. Relative optical densities (ROD) were transformed into levels of bound radioactivity (dpm/mg tissue-equivalent) after calibration using the co-exposed [^14^C]-standard. Regions of interest were then outlined manually with reference to the stereotaxic atlas of Paxinos and Franklin [44]. The cortex was analyzed at three levels: frontal, the level of the striatum, and the level of the hippocampus. Other regions investigated were the cerebellum, thalamus, striatum, hippocampus, and amygdala.

For immunohistochemistry, the right brain hemisphere was fixed, paraffin-embedded and cut into 5 μm coronal sections. For immunohistochemistry sections were de-waxed with xylene and rehydrated by submerging in a graded series of ethanol with decreasing concentrations. Sections to be stained for amyloid-β underwent the following pre-treatment steps. First sections were bleached with potassium permanganate (three min), rinsed in water (one min), decolorized in oxalic acid (one min), and rinsed again (one min). Thereafter, antigen retrieval was performed at room temperature by immersion in formic acid (ten min). Sections to be stained for reactive gliosis and neuronal loss underwent heat-mediated antigen retrieval by microwaving in citrate buffer (pH 6, ten min). For all sections endogenous peroxidase activity was quenched by rinsing in peroxidase blocking solution (DAKO S2023, Glostrup, Denmark) five min). Sections were then incubated with the appropriate primary antibody at room temperature (60 min). For detection of amyloid-β the primary antibody clone 4G8 (Eurogentec SIG-39200, 1/20, 000, Fremont, CA, USA) was used. Reactive gliosis was detected with anti- glial fibrillary acidic protein (GFAP) (Calbiochem IF03L, 1/500, San Diego, CA, USA) and anti-Iba-1 (Wako 019–19741, 1/500 dilution, Richmond, VA, USA) primary antibodies. Following incubation with the respective primary antibody, sections were thoroughly rinsed in wash buffer and subsequently incubated in peroxidase coupled secondary antibody (30 min) (DAKO K4001, DAKO K4002). After, sections were washed in buffer and immunodetection was performed by treatment with the chromogenic 3–3 diaminobenzidine (DAB) solution. Finally sections were counterstained with hematoxylin, dehydrated by submerging in a series of ethanol, fixed in xylene, mounted and coverslipped.

For sections stained for amyloid-β, virtual images were acquired using a Mirax Digital Slide Scanner (Carl Zeiss, Germany) and image analysis was performed using the Definiens analysis software package v1.5. For all other stainings, slides were scanned with a NanoZoomer slide scanner (Hamamatsu Photonics, Shizuoka, Japan) and analyzed with Matlab/Phaedra. In each case, regions-of-interest (ROIs) were manually delineated in accordance with Franklin and Paxinos atlas [44] and for each ROI the percentage of DAB-labeled area was calculated.

### Statistical analysis

Statistical analysis was performed with GraphPad Prism v6 software.

## Results

### *In vivo* μPET imaging

#### [^18^F]FDG

**Fig. 1:**
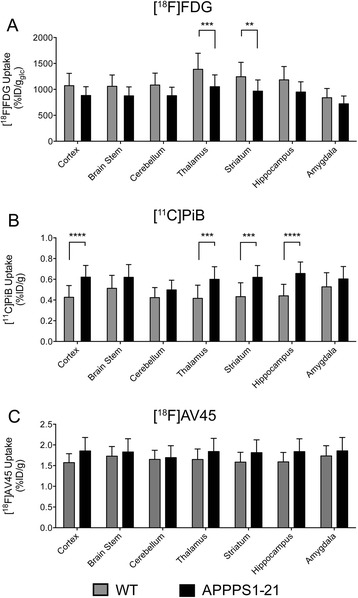
*In vivo* μPET quantification of (**a**) [^18^F]FDG, (**b**) [^11^C]PiB, and (**c**) [^18^F]AV45 uptake in WT and APPPS1-21 mice using VOI analysis. Data are expressed as mean + standard deviation, unpaired Student t-test **** *p* < 0.0001, ***p < 0.001, **p < 0.01, *p < 0.05 (Holm-Sidak correction for multiple comparisons). *μPET* micro-positron emission tomography, *FDG* fluorodeoxyglucose, *PiB* Pittsburgh compound B, *WT* wild type, *VOI* volume of interest

VOI analysis (Fig. 1a) demonstrated a trend of reduced [^18^F]FDG uptake in APPPS1-21 mice that reached significance in the thalamus (*p* = 0.00068) and striatum (*p* = 0.0048). We additionally employed voxel-wise analysis (threshold *p* < 0.01, extent threshold 100 voxels) to investigate whether APPPS1-21 mice had either increased or decreased [^18^F]FDG uptake in comparison to age-matched WT.

**Fig. 2:**
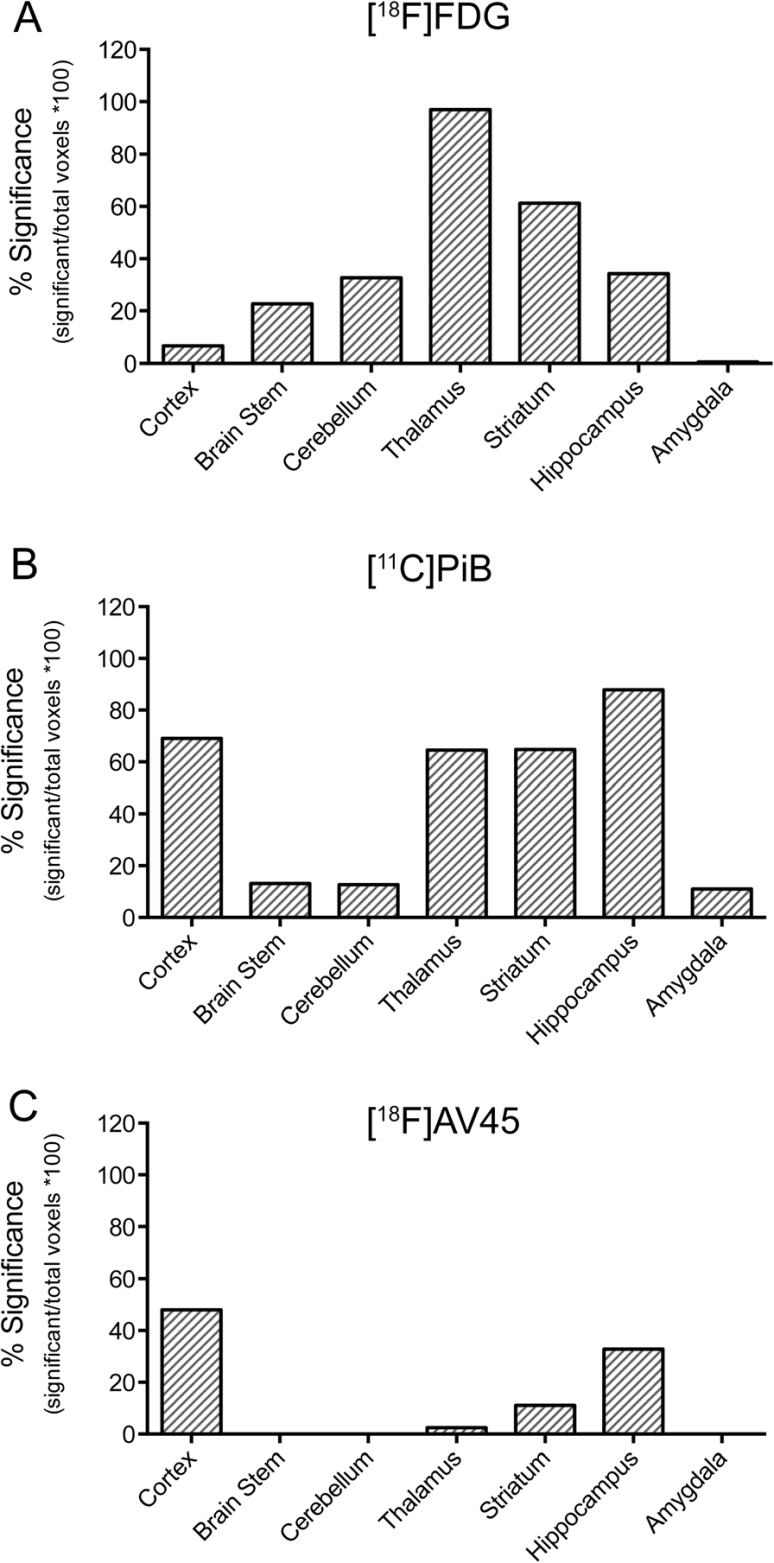
*In vivo* analysis of glucose utilization and fibrillar amyloid-β using voxel-wise SPM analysis. Graphs show the results from regional voxel-wise analysis of μPET data. Statistically significant voxels relative to the total number of voxels in a region are depicted for **a** [^18^F]FDG, **b** [^11^C]PiB, and **c** [^18^F]AV45. ). *SPM* statistical parametric mapping, *μPET* micro-positron emission tomography, *FDG* fluorodeoxyglucose, *PiB* Pittsburgh compound B

**Fig. 3:**
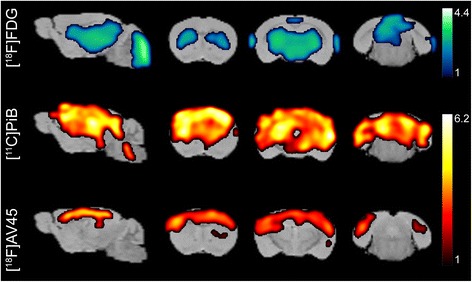
Statistical parametric T-maps obtained from voxel-wise analysis of μPET data with [^18^F]FDG, [^11^C]PiB and [^18^F]AV45

Using two-sample t-tests no voxels were shown to have significantly increased uptake in APPPS1-21 (additionally at the lower threshold of *p* < 0.05). On the other hand, significant decreases(threshold p < 0.01) in uptake were detected and are summarized in Fig. 2a as the percent of significantly changed voxels within each brain region. The thalamus was the most affected with near widespread significant reductions (96.96 %, maxT = 3.35) and was followed by the striatum in which significant decreases covered more than half of the region (61.21 %, maxT = 3.29). The hippocampus (34.22 %, maxT = 3.13), cerebellum (32.68 %, maxT = 3.98), and brain stem (22.66 %, maxT = 4.49) were affected to a lower extent while the cortex (6.74 %, maxT = 3.19) and amygdala (0.57 %, maxT = 2.64) showed minimal changes. This is visualized in Fig. 3 where the statistical T-map illustrates these regions of significantly decreased [^18^F]FDG uptake in APPPS1-21 mice compared to WT.

#### [^11^C]PiB and [^18^F]AV45

VOI analysis (Fig. 1b) demonstrated increased retention of [^11^C]PiB in APPPS1-21 mice with the cortex (*p* = 8.35e-005) and hippocampus (*p* = 1.54e-005) showing the greatest significant difference. [^18^F]AV45 retention was increased in APPPS1-21 mice but did not reach significance.

Additionally, [^11^C]PiB and [^18^F]AV45 uptake was similarly analyzed using voxel-wise analysis and a two-sample unpaired t-test (threshold *p* < 0.01, extent threshold 100 voxels). APPPS1-21 mice demonstrated a number of regions with significantly increased [^11^C]PiB binding. These increases are summarized in Fig. 2b and illustrated in Fig. 3. The hippocampus demonstrated the most substantial changes (87.84 %, maxT = 7.15) followed by the cortex (69.08 %, maxT = 7.93), striatum (64.75 %, maxT = 7.05), and thalamus (64.57 %, maxT = 5.82). Modest changes (<15 %) were observed in the brain stem, cerebellum, and amygdala. Using the same threshold value, significantly increased [^18^F]AV45 uptake was also demonstrated (Fig. 2c); however, in comparison to [^11^C]PiB there were notably lower maxT values (i.e., less significance) and less widespread changes. With this tracer the cortex demonstrated the most widespread significant differences (48.01 %, maxT = 4.68), followed by the hippocampus (32.84 %, maxT = 3.89) and striatum (11.09 %, maxT = 3.59). Figure 3 compares the localization of significant changes for [^18^F]FDG, [^11^C]PiB, and [^18^F]AV45. While amyloid binding is predominantly in the cortex, [^18^F]FDG decreases are in the mid- to hind-brain regions although some overlap can be observed in the thalamus, striatum, and hippocampus.

### *Ex- vivo* validation

#### Ex-vivo [^14^C]2-DG autoradiography

**Fig. 4:**
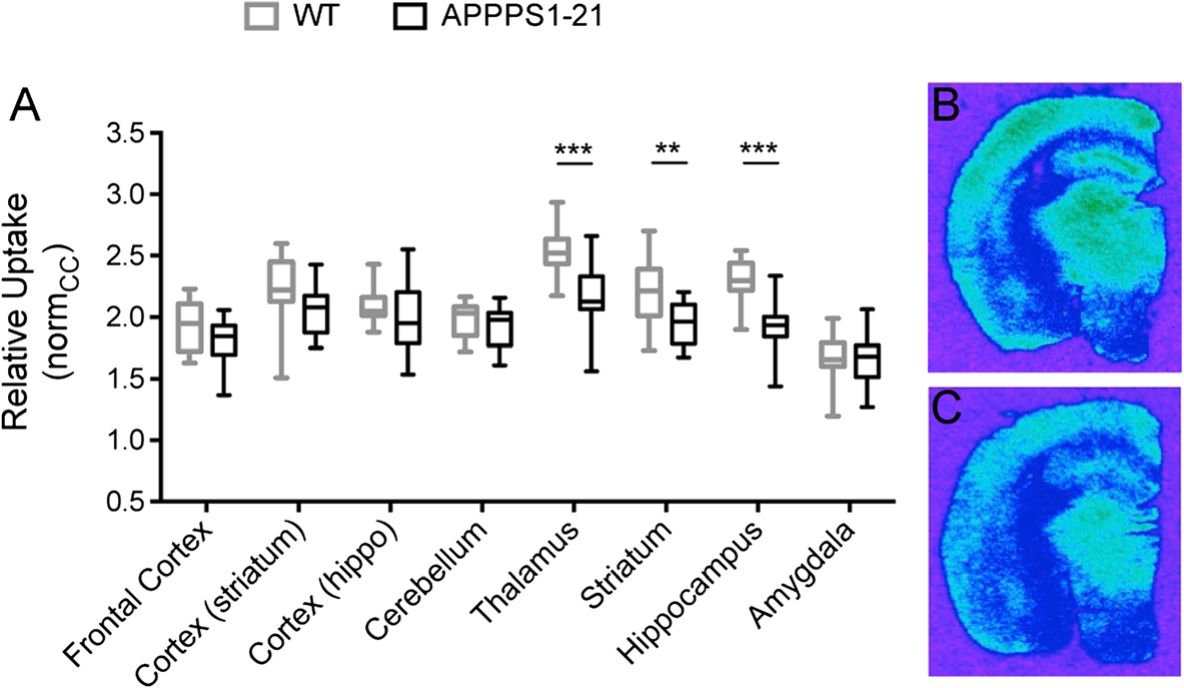
*Ex vivo* measures of glucose utilization with [^14^C]2-DG autoradiography in aged APPPS1-21 and WT mice. **a** Box-plots show the normalized regional [^14^C]2-DG uptake values for WT and APPPS1-21 mice in different brain regions. Unpaired Student t-test,** *p* < 0.01, *** *p* < 0.001. Representative images are shown for (**b**) a WT and (**c**) a APPPS1-21 animal. *2-DG* 2-deoxy-D-glucose, *WT* wild type

Figure 4a shows the quantitative analysis of [^14^C]2-DG autoradiography. The corpus callosum was found to be metabolically stable in APPPS1-21 mice and was thus used as a reference region to normalize regional uptake values. Multiple comparisons using unpaired Student t-tests (with Holm-Sidak correction) were performed for each region to assess the differences between APPPS1-21 and WT mice. This analysis revealed that significant decreases in [^14^C]2-DG uptake were found in the thalamus (*p* = 0.00006), striatum (*p* = 0.0041), and hippocampus (*p* = 0.00006).

#### Immunohistochemistry

**Fig. 5:**
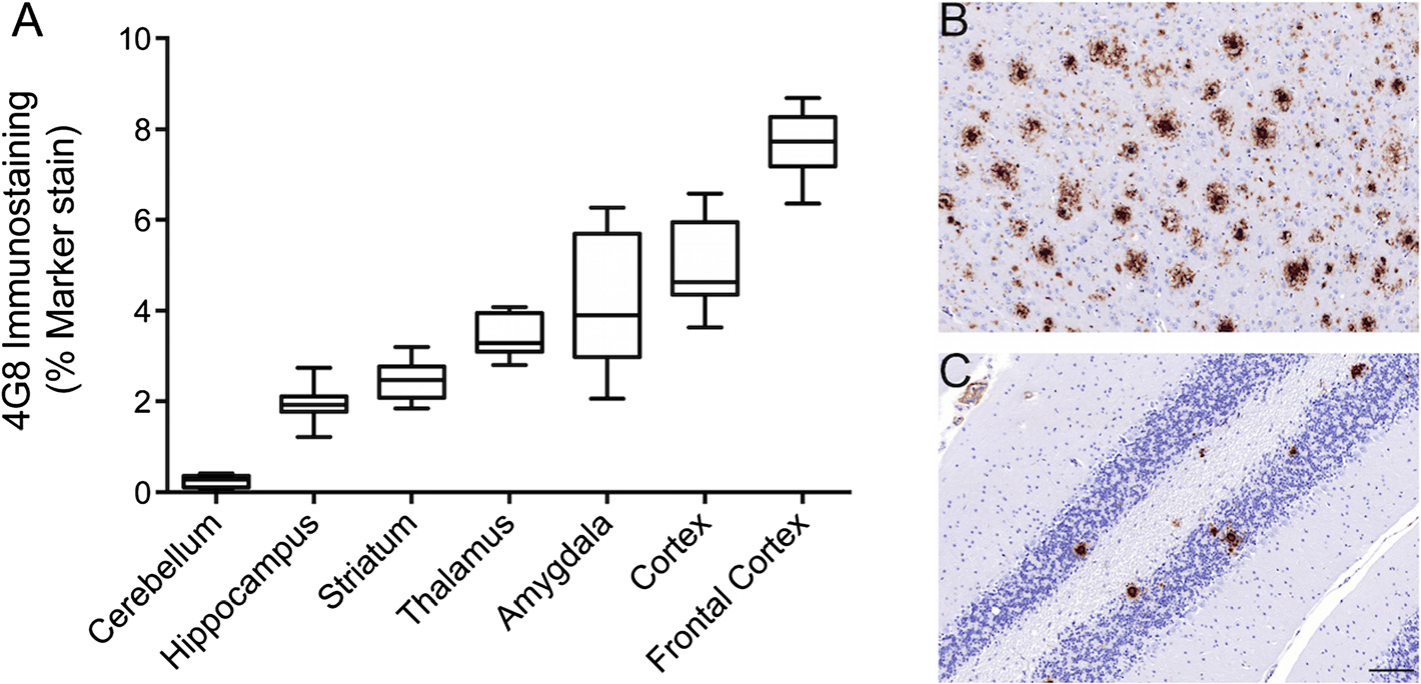
*Ex vivo* immunohistochemistry for amyloid-β with 4G8 immunostaining in APPPS1-21 mice. **a** Data are presented as the max and min ± standard deviation. Representative images from (**b**) the frontal cortex and (**c**) the cerebellum of an APPPS1-21 mouse (Scale bar = 100 μm)

Brain amyloid plaque load was determined in APPPS1-21 mice with 4G8 immunostaining. Plaque load was abundant in these aged mice, with amyloid deposition highest in the frontal cortex (7.67 ± 0.71 % marker stain) followed by the cortex at the level of the hippocampus (4.95 ± 0.97 % marker stain), amygdala (4.12 ± 1.47)  and thalamus (3.43 ± 0.45 % marker stain). In line with its low transgene expression, minimal plaque staining was observed in the cerebellum (0.24 ± 0.14 % marker stain) (Fig. 5c). No amyloid plaques were detected in WT mice.

**Fig. 6:**
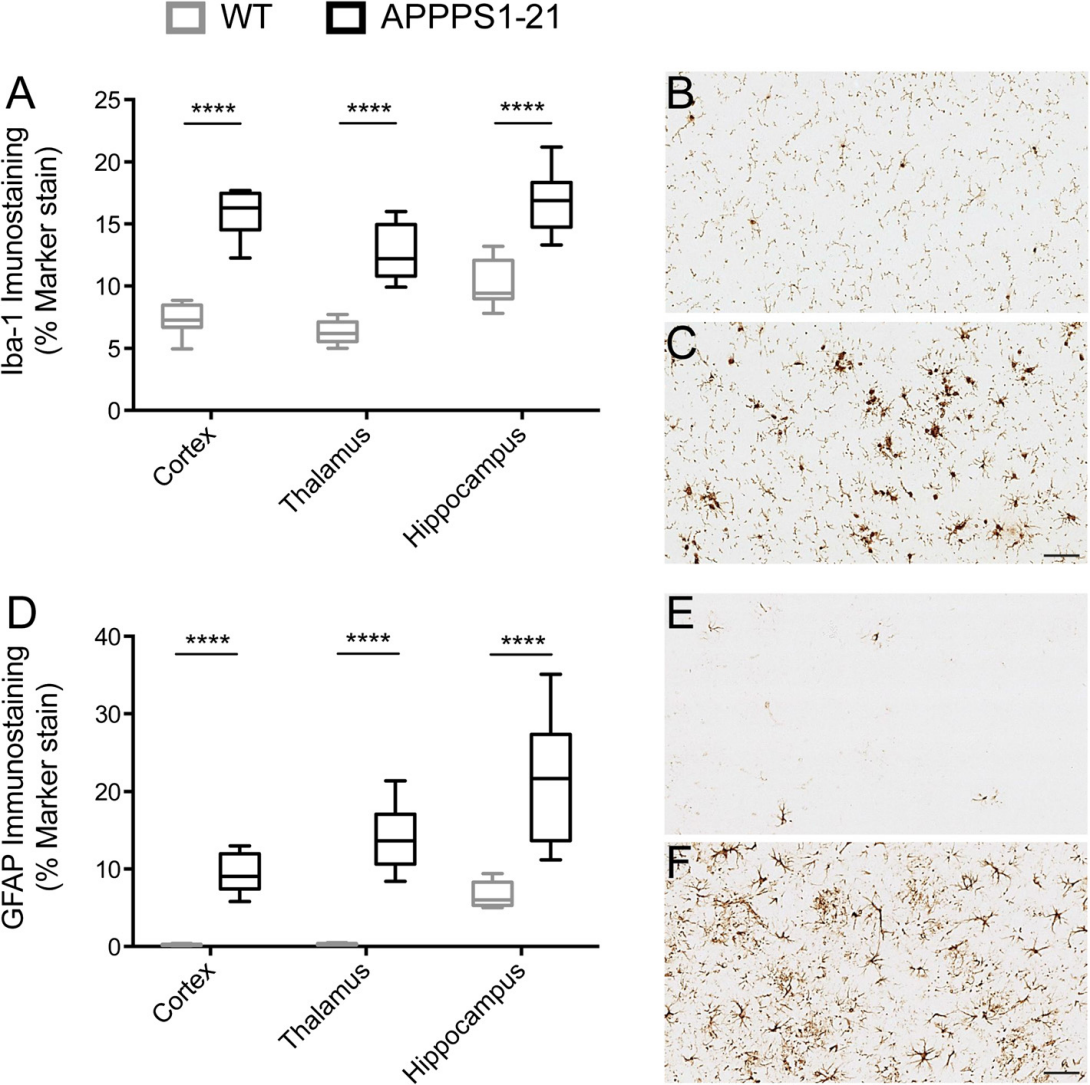
*Ex vivo* immunohistochemistry for the inflammatory markers GFAP and Iba-1 in APPPS1-21 and WT mice. **a** and (**d**) data are presented as the max and min ± standard deviation. Unpaired Student t-test **** *p* < 0.0001. Representative images from the cortex of WT (**b**) and (**e**), and APPPS1-21 **c** and **f** mice (Scale bar = 50 μm). *GFAP* glial fibrillary acidic protein, *Iba-1* ionized calcium-binding adaptor molecule 1, *WT* wild type

Genotypic differences in inflammatory markers were investigated in the cortex, hippocampus, and thalamus by immunohistochemistry for activated microglia and astrocytes with Iba-1 (Fig. 6a-c) and GFAP, respectively (Fig. 6d-f). Multiple comparisons using unpaired Student t-tests (with Holm-Sidak correction) were performed for each region to assess the differences between APPPS1-21 and WT mice. Both GFAP and Iba-1 staining were significantly increased in APPPS1-21 mice in the regions investigated (*p* < 0.0001) (Fig. 6a and d).

### Correlation analysis

**Fig. 7:**
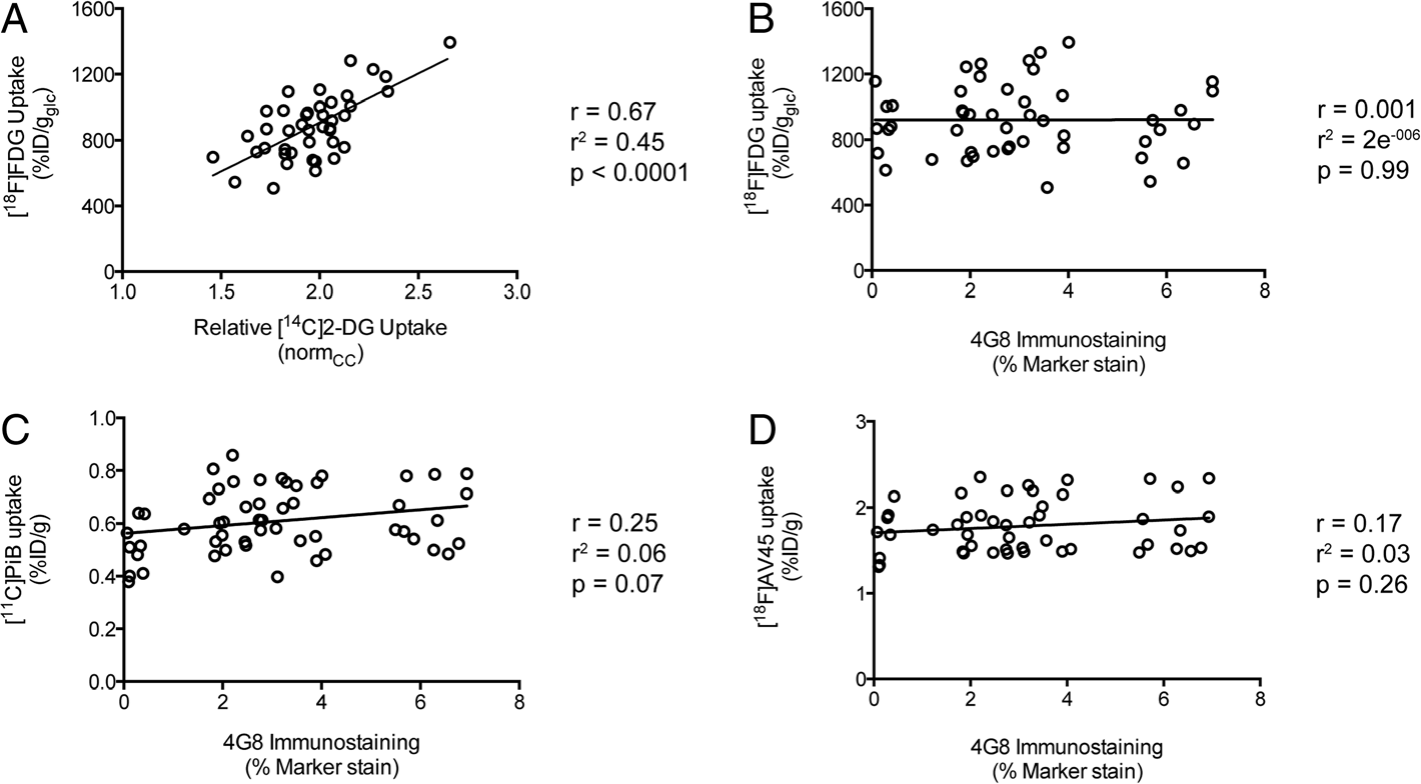
Correlation analyses between *in vivo* μPET data and *ex vivo* measures of amyloid burden and glucose utilization. **a** Graph depicting [^18^F]FDG and [^14^C]2-DG quantification of brain glucose utilization. **b** Graph of [^18^F]FDG uptake versus amyloid burden. **c** Graph of [^11^C]PiB and 4G8 quantification of amyloid burden. **d** Graph of [^18^F]AV45 and 4G8 quantification of amyloid burden. Each dot represents a regional value from an individual animal (cortex, striatum, amygdala, hippocampus, thalamus, and cerebellum). Only APPPS1-21 mice were included in this analysis. *μPET* micro-positron emission tomography, *FDG* fluorodeoxyglucose, *2-DG* 2-deoxy-D-glucose, *PiB* Pittsburgh compound B

To investigate the relationship between the *in vivo* μPET measurements and relative *ex vivo* gold standards in APPPS1-21 mice we performed correlation analyses (Pearson correlation test). The absolute quantification of the μPET data in either %ID/g ([^11^C]PiB and [^18^F]AV45) or %ID/g_glc_ ([^18^F]FDG) derived from VOI analysis (Fig. 1) was employed in this analysis. We found that regional [^18^F]FDG uptake values demonstrated a significant positive correlation to *ex vivo* [^14^C]2-DG measures in these transgenic mice (r = 0.67, r^2^ = 0.45, *p* <0.0001) (Fig. 7a). We investigated the association between regional [^18^F]FDG uptake and amyloid-β deposition but found that these measures were not correlated (r = 0.01, r^2^ = 2e^−006^, *p* = 0.99) (Fig. 7b). In contrast, *in vivo* [^11^C]PiB and [^18^F]AV45 binding did not correlate strongly to *ex vivo* 4G8 immunostaining for amyloid-β in APPPS1-21 mice (r = 0.25, r^2^ = 0.06, *p* = 0.07 and r = 0.17, r^2^ = 0.03, *p* = 0.26 respectively) (Fig. 7c and d).

## Discussion

Due to its non-invasive nature, μPET imaging is an attractive modality for preclinical investigation that allows for the visualization of multiple neuropathological biomarkers. [^18^F]FDG detected significant decreases in cerebral metabolism in APPPS1-21 mice and showed a high congruency to *ex vivo* measures of [^14^C]2-DG. Both [^11^C]PiB and [^18^F]AV45 demonstrated significant uptake in APPPS1-21 mice but showed poor sensitivity when compared to *ex vivo* amyloid quantification. The detected reductions in [^18^F]FDG did not correlate to regions of highest amyloid-β burden.

A number of animal models of AD have been investigated with *in vivo* [^18^F]FDG imaging but no consensus has been reached on its utility. On the whole, most investigations of amyloidosis-only models have revealed unchanged [24, 25] or increased uptake [26–28], counter to the clinical phenotype. These findings were interpreted as a fundamental limitation of animal models and, additionally, a limit in the sensitivity of μPET. However, more recently, widespread significant decreases in [^18^F]FDG in the 5 x FAD model have been reported [31] and we have detected significant regional decreases in the double transgenic TASTPM model [32, 33]. It is possible that normal, hypometabolism and hypermetabolism can be observed at different disease stages in animal models depending on disease severity that depends on both the mutations and the age at imaging. Other likely contributors to the controversy are methodological differences, especially in relation to analysis. Normalization of regional uptake values to a reference region is common but may yield inaccurate results if such regions are compromised by disease [45]. In comparison the use of voxel-wise analysis provides more sensitivity to detect changes and allows for multiple hypothesis testing (i.e., hyper or hypo) and should thus be routinely implemented in studies of cerebral glucose metabolism One limitation of μPET imaging is the necessitation to anesthetize the animal and in the case of [^18^F]FDG studies this results in substantially reduced brain uptake of the tracer [46]. The [^14^C]2-DG protocol is, however, performed without the confounding effects of anesthesia and, thus, the correlation between *in vivo* and *ex vivo* results strongly suggests that the [^18^F]FDG findings are reliable. With the aforementioned methodological considerations we have now demonstrated [^18^F]FDG reductions in two transgenic models of AD and aim to further extend our findings in other models for multiple stages of pathology.

While this is the first investigation of cerebral glucose utilization in the APPPS1-21 model, these mice have been previously characterized with amyloid imaging [15, 16, 38]. Significant differences between WT and APPPS1-21 mice in the cortex have been detected at five months with [^18^F]AV45 [16] and at 8.4 months with [^11^C]PiB [15]. In this study, both [^11^C]PiB and [^18^F]AV45 demonstrated increased retention in APPPS1-21 mice with [^11^C]PiB showing a higher discriminative power. [^11^C]PiB has previously been shown to have a greater dynamic range than most fluorinated alternatives [38, 47]. The reduced contrast obtained with tracers, such as [^18^F]AV45 and [^18^F]florbetaben, is due to a higher proportion of non-specific binding resulting from a combination of white matter retention and the uptake of brain penetrant radiometabolites. With relevance to the current findings, Choi et al. described two main brain penetrant radiometabolites of [^18^F]AV45 [48]. The non-specific uptake of these metabolites most likely accounts for the poor performance of [^18^F]AV45 when compared to [^11^C]PiB. A previous preclinical study found a similarly low contrast whereby only a 14.5 % difference between WT and transgenic mice (5XFAD) was observed with [^18^F]-AV45 in comparison to a 21 % difference obtained with [^11^C]-PiB in the same mouse cohort [27]. While we detected amyloid-β *in vivo* with [^11^C]PiB and [^18^F]AV45 binding, we observed discrepancies with *ex vivo* determinants of amyloid-β load for both these tracers. Voxel-wise analysis of [^11^C]PiB binding found the hippocampus to have the most widespread increases whereas immunohistochemistry showed this region had lower amyloid-β deposition than in the thalamus, cortex, and striatum. We have previously described poor correlation between *in vivo* binding of amyloid tracers and *ex vivo* measures of amyloid with [^18^F]AV45 [33], [^18^F]florbetaben, and [^11^C]PiB [38]. The accuracy of *in vivo* detection could be improved by partial volume corrections [49] but the poor correlation is also indicative of the high non-specific binding inherent to these amyloid tracers.

Although aged APPPS1-21 mice demonstrated significant reductions in cerebral metabolism, the regions affected were not congruent with those of human AD. In AD, patients typically show reduced [^18^F]FDG uptake in the precuneus, posterior cingulate, and temporal and parietal cortex. With increasing disease severity hypometabolism spreads to frontal association cortices but metabolism remains largely preserved in the striatum, thalamus, visual and sensorimotor cortices, and the cerebellum and brain stem throughout disease progression [2]. In aged APPPS1-21 mice, minimal reductions were observed in the cortex whereas the most significant reductions were in the thalamus and striatum. Moreover, typically amyloid-free regions in these mice, such as the brain stem and cerebellum, also demonstrated significant reductions. While some previous *ex vivo* autoradiography studies with [^18^F]FDG and [^14^C]2-DG found significant decreases in AD relevant structures, such as the retrospinal and/or cingulate cortex [19, 20], the overall pattern of [^18^F]FDG/[^14^C]2-DG was incongruent to human AD. These incongruences are perhaps not surprising given the inherent differences between animal models and human AD. The majority of cerebral amyloidosis models do not develop overt neuronal death [9] which is a prominent hallmark of human AD and is thought to largely underlie decreases in [^18^F]FDG uptake. While amyloid deposition initiates at six to eight weeks of age, only modest neuronal loss is observed beginning at eight months in APPPS1-21 mice [36]. Accordingly, these animals showed no brain atrophy when investigated with MRI voxel-based analysis [50]. The neuronal loss that was observed was found in the dentate gyrus and CA1 layers of the hippocampus [36, 51]. Atrophy and neuronal loss were notably absent in the cortex which could explain the lack of [^18^F]FDG decreases in this region [50, 51]. Further investigations into the biological underpinnings of [^18^F]FDG decreases in this model are required. Of interest, early loss of dendritic spines has been reported in APPPS1-21 mice and could thus represent a relevant target for future studies [34].

Despite some overlapping pathology, decreases in [^18^F]FDG uptake do not correlate well with increases in amyloid retention [52, 53]. Inflammation in high plaque-bearing regions has been postulated as a factor for the lack of correlation between amyloid tracer retention and reductions in [^18^F]FDG [54]. As glial cells also use glucose as an energy source it is proposed that increased inflammation in proximity to plaques may mask decreased neuronal activity. Indeed, in a study of aged monkeys [^18^F]FDG uptake positively correlated with increased binding of the microglial marker [^11^C]DPA-713 rather than with [^11^C]PiB binding [54]. In APPPS1-21 mice, activated microglia and astrocytes appear in tandem with the onset of amyloid deposition and increase in number with disease progression [36]. In line with these observations, we demonstrated significant increases in inflammatory markers for astrocytes (GFAP) and microglia (Iba-1) in the thalamus, cortex and hippocampus of APPPS1-21 mice. Despite this robust neuroinflammation, APPPS1-21 mice still demonstrate significant [^18^F]FDG decreases. While this does not discount the involvement of inflammation in the [^18^F]FDG signal, taken together we find it insufficient to fully account for the poor correlation. An alternative hypothesis for this dissociation is network degeneration which proposes that the disease propagates along neuronal networks [55]. In this case amyloid deposition in addition to causing local synaptic dysfunction would also induce dysfunction in functionally connected regions which may themselves be amyloid-free. This theory is supported by recent clinical evidence demonstrating that worsening amyloidosis is correlated to worsening hypometabolism in both local and functionally related regions [56]. It should, however, be remembered that imaging data relates to measures of fibrillar amyloid-β and thus does not provide information on the relationship between soluble amyloid-β which is especially neurotoxic and likely influences neuronal dysfunction [57]. Interestingly, decreases in functional connectivity have been reported in APPPS1-21 mice with resting state fMRI [58].

We only investigated pathology in our animal model at a single time point. Additionally we choose this old age time point as we expected substantial pathological burden at this stage of disease. Therefore, to fully assess the utility of [^18^F]FDG and to delineate the temporal relationship between amyloid deposition and neuronal dysfunction a longitudinal study should be performed. Furthermore, in this study we aimed to primarily demonstrate [^18^F]FDG as a useful tracer in preclinical investigations. Future investigations should focus on finding the underlying mechanisms of these [^18^F]FDG alterations and their relationship to neurodegeneration as has been recently described in a Tau transgenic model [59].

## Conclusions

Our study provides support for the use of [^18^F]FDG as an *in vivo* technique to investigate abnormal cerebral glucose utilization in transgenic models of AD. Our findings suggest that the combined use of *in vivo* amyloid and [^18^F]FDG μPET imaging will aid in understanding the relationship between amyloid and neuronal dysfunction.

## Abbreviations

%ID/g: Percent injected dose per gram
%ID/g_glc_: Percent injected dose per gram glucose corrected
2-DG: 2-Deoxy-D-glucose
AD: Alzheimer’s Disease
ANOVA: Analysis of variance
APP: Amyloid precursor protein
CT: Computed tomography
DAB: 3,3-Diaminobenzidine
dpm: Disintegrations per minute
FDG: Fluorodeoxyglucose
FORE: Fourier rebinning
FWHM: Full width at half maximum
GFAP: Glial fibrillary acidic protein
Iba-1: Ionized calcium-binding adaptor molecule 1
keV: Kiloelectron-volt
maxT: Maximal T-value
MBq: Megabequeral
MR: Magnetic resonance
MRI: Magnetic resonance imaging
NeuN: Neuronal nuclei
nsec: Nanosecond
OSEM2D: Two-dimensional ordered subset estimation maximization
PET: Positron emission tomography
PiB: Pittsburgh compound B
PS1: Presenilin 1
ROD: Relative optical density
ROI: Region of interest
SPM: Statistical parametric mapping
SSS: Single scatter simulation
uA: Microampere
VOI: Volume of interest
WT: Wild type
μPET: Micro-positron emission tomography
